# A Review on Sustainable Inks for Printed Electronics: Materials for Conductive, Dielectric and Piezoelectric Sustainable Inks

**DOI:** 10.3390/ma16113940

**Published:** 2023-05-24

**Authors:** Leire Sanchez-Duenas, Estibaliz Gomez, Mikel Larrañaga, Miren Blanco, Amaia M. Goitandia, Estibaliz Aranzabe, José Luis Vilas-Vilela

**Affiliations:** 1 Surface Chemistry & Nanotechnologies Unit, Fundación Tekniker, Inaki Goenaga 5, 20600 Eibar, Spain; estibaliz.gomez@tekniker.es (E.G.); miren.blanco@tekniker.es (M.B.); amaia.martinez@tekniker.es (A.M.G.); estibaliz.aranzabe@tekniker.es (E.A.); 2 Electronics and Communications Unit, Fundación Tekniker, Inaki Goenaga 5, 20600 Eibar, Spain; mikel.larranaga@tekniker.es; 3 Department of Physical Chemistry, Faculty of Science and Technology, University of the Basque Country, UPV/EHU, Barrio Sarriena s/n, 48940 Leioa, Spain; joseluis.vilas@ehu.eus

**Keywords:** printed electronics, sustainable materials for printed electronic inks, conductive ink, dielectric ink, piezoelectric ink, biobased ink, biodegradable ink, sustainable ink

## Abstract

In the last decades, the demand for electronics and, therefore, electronic waste, has increased. To reduce this electronic waste and the impact of this sector on the environment, it is necessary to develop biodegradable systems using naturally produced materials with low impact on the environment or systems that can degrade in a certain period. One way to manufacture these types of systems is by using printed electronics because the inks and the substrates used are sustainable. Printed electronics involve different methods of deposition, such as screen printing or inkjet printing. Depending on the method of deposition selected, the developed inks should have different properties, such as viscosity or solid content. To produce sustainable inks, it is necessary to ensure that most of the materials used in the formulation are biobased, biodegradable, or not considered critical raw materials. In this review, different inks for inkjet printing or screen printing that are considered sustainable, and the materials that can be used to formulate them, are collected. Printed electronics need inks with different functionalities, which can be mainly classified into three groups: conductive, dielectric, or piezoelectric inks. Materials need to be selected depending on the ink’s final purpose. For example, functional materials such as carbon or biobased silver should be used to secure the conductivity of an ink, a material with dielectric properties could be used to develop a dielectric ink, or materials that present piezoelectric properties could be mixed with different binders to develop a piezoelectric ink. A good combination of all the components selected must be achieved to ensure the proper features of each ink.

## 1. Introduction

Printed electronics are limited by parameters such as their cost, maintenance, or the production of significant quantities of electronic waste. In the last decades, due to the increasing demand for electronics and the speed-up of programmed obsolescence, there has been an increase in the quantity of produced electronic waste (e-waste) [1]. Moreover, the materials used to produce printed electronics are usually metals, such as gold, silver, palladium, tin, or copper, because of their chemical stability and non-degradability. Using metallic materials in printed electronics ensures an appropriate electrical behavior, showing electrical conductivities up to 10^7^ S/m, while other materials, such as carbon, present electric conductivities up to 10^5^ S/m. Nevertheless, this represents a high consumption of precious metals, as well as a problem for society, as some of them are considered critical raw materials (CRMs) [2]. The 2023 EU list of CRMs is shown in Table 1 [3].

**Figure 1:**
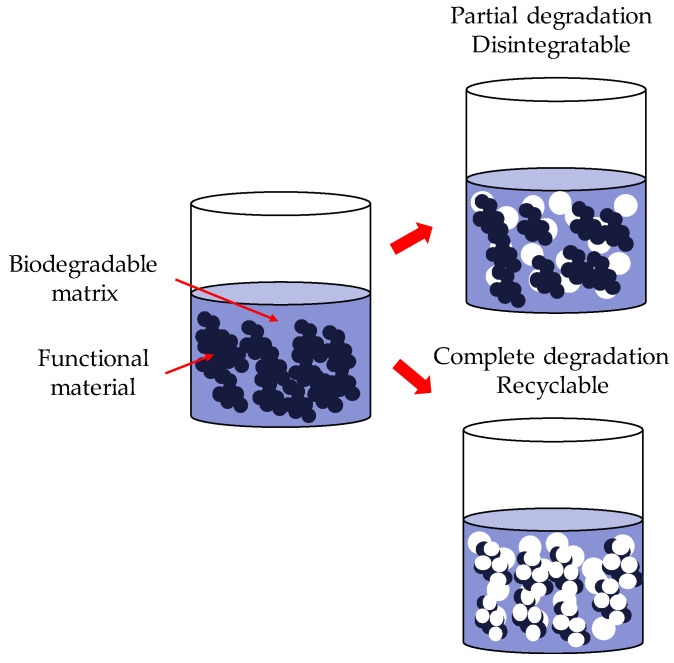
Scheme of the processes of a disintegrable system.

Therefore, there is a need to develop more environmentally friendly electronic systems. This need could be achieved in two different ways: by using sustainable or biobased materials or by using biodegradable and/or recyclable materials. Sustainable or biobased materials come from a living matter source (biomass) and reduce the environmental impact. Biodegradable and/or recycled materials can degrade partially or completely after their service life, as shown in Figure 1 [4].

However, the development of biodegradable electronics (commonly called green electronics) with high conductivity manufactured using printing technologies in environmental conditions is currently a challenge [5,6].

There are available devices with conductive lines (such as magnesium, zinc, or iron, which are degradable in physiological environments, in certain conditions of pH and temperature). The conductive lines are deposited in a biodegradable substrate (sodium carboxymethyl cellulose, silk, etc.) and manufactured using chemical or physical vapor deposition [7]. These devices are designed to degrade in a water-based solution, making them inappropriate for applications in environments with considerable humidity [6].

To consider printed electronic devices sustainable, the substrate and the ink deposited on them should also be sustainable. Examples of various sustainable substrates are rice paper, biodegradable polymers such as PLA (polylactic acid) or its derivates, silk, or cellulose paper, among others [7,8]. Moreover, the used deposition technique must be environmentally friendly, making inkjet printing a good deposition technique because it wastes little material [9].

Most of the inks employed to produce electronic devices using inkjet printing are a combination of organic polymers and functional materials, depending on the final characteristics of the device [10].

**Figure 2:**
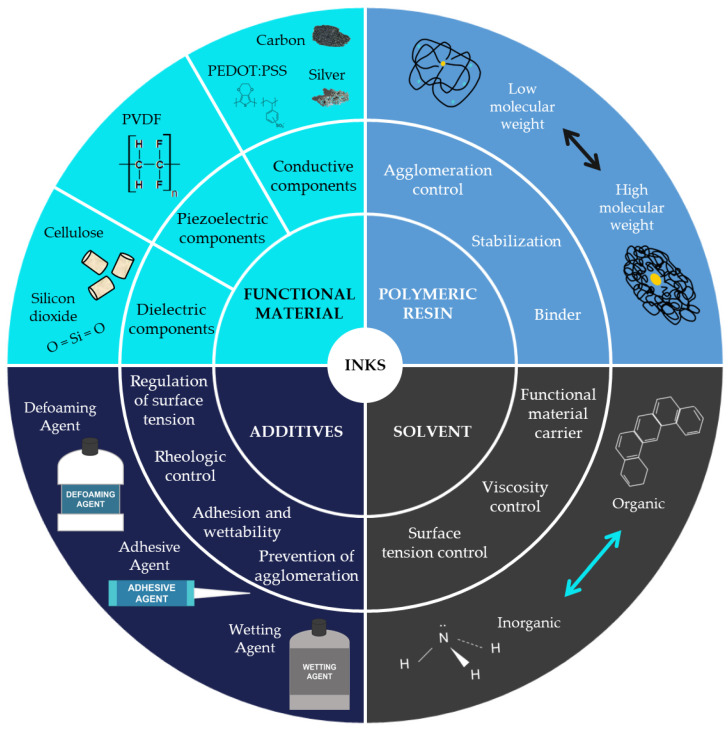
Ink composition.

The most common classification of inks for printed electronics is based on their electrical properties. This classification includes conductive, semi-conductive, dielectric, and resistive inks. Nevertheless, as this review tries to collect the materials of the inks, the selected classification focuses on the ink’s composition. Taking this into account, it is possible to define four main components of an ink, as shown in Figure 2.

1. Functional material

The functional material is responsible for giving the ink its properties. Depending on the ink’s final functionality, it is possible to classify inks into three groups: conductive inks, dielectric inks, and piezoelectric inks. Conductive inks are those that, after being processed, have electrical properties. They are composed of materials such as metals (for example, silver particles), carbon derivates, or conductive polymers. Dielectric inks are formulated to have the property of isolating the place where they are deposited. The used materials could be cellulose or silicon dioxide, among others. Piezoelectric inks can produce an electrical current when suffering a slight deformation or being deformed when an electrical current is applied. Their composition materials must exhibit this property, such as the PVDF.

2. Solvent

The functional material is dispersed in a solvent and then mixed with the polymeric resin.

3. Polymeric resin or binder

The polymeric resin acts like the binder of the ink, being responsible for the ink’s mechanical properties, its stability, or avoiding particle agglomeration.

4. Additives

The last component of the inks is the additive, used to improve or secure certain properties of the mixture, such as controlling the surface tension, its rheological properties, its adhesion and wettability, or preventing agglomeration [11].

All these elements of ink’s composition have to be used in the correct proportion to ensure the functionality of the ink and its properties. These properties, such as the solid content of the ink, may vary depending on the deposition method used. For example, in technologies such as inkjet printing, liquid inks are needed. The viscosity of these types of inks is lower than pastes’ viscosities, which are designed to be deposited by screen printing or similar methods. To reach the specific properties of each type of ink, the specific components must be adjusted. The solid content of each ink will vary depending on the desired final properties as well as the functional material used. The viscosity of the ink tends to increase with increasing solid content. Inks with solid contents varying between 20% and 80% have been found. The content of additives in an ink is usually less than 5–10%, and the remaining content is a combination of a polymeric resin and the solvent where the functional material is dispersed [12,13,14]. Table 2 shows the percentages of each component of screen printing and inkjet printing inks.

To ensure the sustainability of the formulated inks, it is important to select the materials that make up most of the ink.

In this review, the different functional materials that can be used to develop sustainable inks, as well as the solvents and binders used, are collected. Additives, being a small percentage of the whole ink, are not analyzed.

To be considered sustainable, the formulated ink must follow one of these criteria:

1. It is a biobased ink. Most of its compounds are produced from a natural or renewable source. Some examples are materials produced from biomass (produced from agricultural waste), obtained from a biogenic process, or produced using a sustainable route, among others. As with biobased plastics, currently, there is no rule that measures the sustainability of an ink based on its biobased content [15].
2. It is a biodegradable ink. Most of its compounds degrade partially or totally in a reasonable period. For polymers, the degradation is measured by the UNE-EN ISO 14855 rule [16], which measures the biodegradation of the polymer in ambient compost, or the UNE-EN ISO 14852 [17], measuring the biodegradability in aqueous media, at 20–25 °C for 6 months.
3. Its compounds are not considered critical raw materials or harmful to the environment.

**Figure 3:**
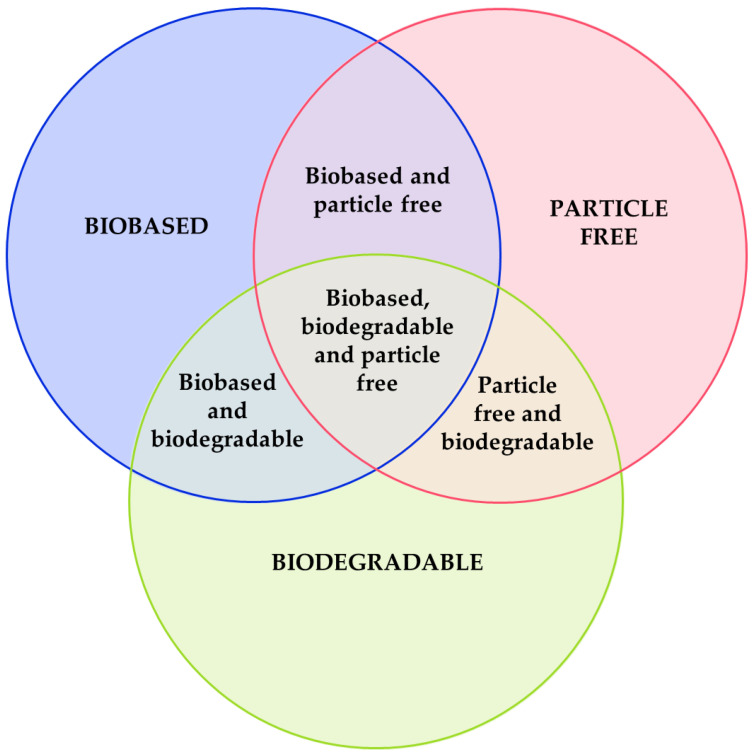
Criteria to classify a sustainable ink.

The resultant ink could be also a combination of these criteria, as shown in Figure 3. Each material used, if sustainable, could be classified in a section of Figure 3. An aspect to consider when developing sustainable inks is the fabrication and material costs. If the developed ink is extremely expensive, it would not be competitive with commercial ones. Being too expensive prevents these types of inks from achieving their main objective: to reduce electronic waste.

## 2. Composition of Inks

### 2.1. Functional Material

#### 2.1.1. Conductive Inks

Conductive inks are generally composed of a functional material (or its precursor), which gives the ink its electrical conductivity properties. If the ink is composed of the precursor, the metal particles are prepared with bottom-up methods, decomposing the precursor’s molecules thermally or through the reduction of metal salts reacting with a reduction agent. Depending on the functional material used, conductive inks could be classified into three separate groups: (1) conductive inks formed of metallic particles, (2) carbon-based conductive inks, or (3) particle-free conductive inks.

Most conductive inks use metallic materials, such as silver or copper, to achieve good electric conductivity. However, some of the used materials are considered critical raw materials or are harmful to some species (such as silver for submarine life), entailing a risk to the environment [18].

Carbon-based materials are the second family of materials used to give inks electrical conductivity. These materials have demonstrated good conductivity and can come from an inexhaustible source. Their biodegradability is secured in certain conditions; for example, carbon nanotubes (CNTs) degradation is produced by macrophages [19]. These allotropic forms of carbon (graphite, graphene, CNTs, or carbon black) can be used separately or by combining their properties to ensure good conductivity. Carbon is an element present in nature that can be found in fossil form, in the air, or in the ocean, and can be processed and afterward recycled and returned to nature.

The third family of inks contains those that do not have particles, such as Poly(2,3-dihydrothieno-1,4-dioxin)-poly(styrenesulfonate) inks (PEDOT:PSS inks). This material is not biodegradable at all, but, used in a small proportion, the final ink could be considered biodegradable.

Depending on the selected material, the conductivity of the final ink will vary. Table 3 contains the values of conductivity of various functional materials.

A strategy to synthesize biogenic silver particles has been developed in recent years. This sintering method is low cost, causes less toxic waste, and consumes less energy while a higher yield is obtained. The process is based on the ability of certain organisms, such as bacteria, yeasts, or fungi, to alter the chemical nature of metals and reduce them into nanoparticles [24]. Depending on the specific organism used and the environmental conditions, the nanoparticles obtained may have different physicochemical properties [24].

Nevertheless, the attainment of silver nanoparticles depends on numerous factors, such as the genetic properties of the organisms or the environmental conditions [11]. If sintering silver through this process, we could consider it a biobased material [25]. Even so, silver is a problem for submarine life and is not biodegradable, becoming a problem for the environment [18].

Carbon can be provided from biomass produced from agricultural waste. This source is abundant, sustainable, renewable, and rich in this material (up to 55% of biomass is carbon). It is necessary to apply thermal treatments to biomass to obtain graphitic structures [26].

Other carbon sources are vegetable oils, chicken oil, or camphor (C_10_H_16_O) after being correctly processed [27,28,29,30]. Carbon nanostructures could also be formed through the thermal treatment of cellulose using a nickel salt during the process [31].

It has been demonstrated that graphene could be obtained from daily materials such as food, waste, plastics, or plants, treating them at hot temperatures in an H_2_/Ag atmosphere to obtain high-quality graphene layers [30].

CNTs are generally obtained through the Chemical Vapor Deposition (CVD) process. The sustainability of this process can be improved by using more sustainable catalysts or renewable carbon sources. Metals or metallic oxides that can be found in nature, such as lava or sand, could be used as catalysts [32,33]. These sources are not commonly considered renewable but are abundant in nature and low cost. However, the use of these catalysts does not lead to uniformity in the morphology or the nanostructure of the CNTs [30]. CNTs could also be produced using iron extracted from plants such as sesame seeds as organic precursors [34]. The obtained CNTs present a uniform size [35]. Pol et al. described a process to obtain CNTs from polymer waste without solvents [36]. This process is based on a thermal dissociation in a closed system within autogenic pressure and catalysts. The procedure described could, in addition, solve another current environmental problem: plastic degradation [30].

Focusing on the biodegradability of carbon-based materials, there are studies that demonstrate the degradation of CNTs. This degradation occurs not only chemically with strong oxidants or thermal treatments in an oxygen atmosphere but also through enzymatic oxidation with horseradish peroxidase. These studies demonstrate the total degradation of the CNTs in in vitro systems (using different animal tissues, cells, or molecules) without cytotoxicity. However, for in vivo systems (evaluating the degradation of CNTs in living organisms), the degradation that occurs is partial, and there is still long-term concern about its toxicity [19].

Taking all this into account, we can classify carbon as biobased and a material that could be biodegradable in certain conditions.

Other metals that are considered to be biodegradable, such as magnesium (Mg), zinc (Zn), or iron (Fe), can be used to develop conductive inks [7]. These are corrodible metals, and they degrade relatively quickly. Mg and Zn are more often used due to their lower cost and ease of processing. However, they degrade more quickly than Fe, making the last one more suitable for applications with a longer lifetime [7]. Lee et al. developed bioresorbable systems using Zn microparticles sintered electrochemically. The substrate used to deposit them was a bioresorbable polymer: a sheet of polylactic-co-glycolic acid (PLGA) [37]. Hwang et al. manufactured biodegradable electronics using Mg, a biodegradable polymer, among other materials [38].

The last type of conductive inks found in the market are those composed of a conductive polymer, such as PEDOT:PSS. Their conductivity is lower than particle-based ones, but it could be interesting to study the possibility of developing a conductive ink based on a biopolymer. PEDOT:PSS is a conductive polymer exhibiting biocompatibility, electrochemical properties, good electric conductivity, and versatile processing, and it is commercialized in water dispersion. There are studies demonstrating the biodegradability of montmorillonite/PEDOT:PSS composites (MMT/PEDOT:PSS) after being processed by specific super worms [39]. PEDOT:PSS does not fulfill the biodegradability ISO 14852 rule [9], which measures its degradation in aqueous media. Nevertheless, Pietsch et al. developed PEDOT:PSS biodegradable electrodes according to the ISO 14855 rule, which measures de degradation of the complete system in compost media [9]. This happens because the quantity of the PEDOT:PSS in the device is less than the non-degradable quantity accepted by the ISO 14855 rule to consider it biodegradable. Therefore, to consider a PEDOT:PSS ink biodegradable, it is necessary to combine a low quantity of PEDOT:PSS with an appropriate biodegradable binder [40]. The main strategy for using the PEDOT:PSS as functional material is to mix it with a biodegradable polymer, keeping conductivity. Conductivities up to 4 7 × 10^−1^ S/m have been reached in particle PEDOT systems dispersed in poly(L-lactic acid) (PLLA) [41]. Mantione et al. developed different PEDOT:biopolymer dispersions, achieving conductivities up to 7 × 10^2^ S/m. The different PEDOT:biopolymer dispersions and the conductivities achieved are collected in Table 4 [42]. These types of inks are particle-free inks.

#### 2.1.2. Dielectric Inks

Dielectric inks are insulator inks below a certain electric voltage, called the breakdown voltage. Therefore, dielectric inks should be formulated with insulator materials that are biobased or biodegradable and mixed with the appropriate solvents, binders, and additives, as shown in Figure 2.

Dielectric inks define their isolation capacity depending on their dielectric constant. The dielectric constant of materials usually used in electronics is shown in Table 5.

Hereinafter, different biobased and/or biodegradable materials that exhibit dielectric properties and are good candidates for formulating dielectric inks are collected.

There are biodegradable inorganic dielectric materials, such as silicon dioxide (SiO_2_), magnesium oxide (MnO), or silicon nitride (Si_3_N_4_), that have been used as dielectric materials due to their dielectric properties [7]. SiO_2_ has a dielectric constant of 3.9, while the dielectric constant of Si_3_N_4_ is 7.5 [48]. This type of material could be dispersed in a binder to develop a dielectric ink for printed electronics.

**Figure 4:**
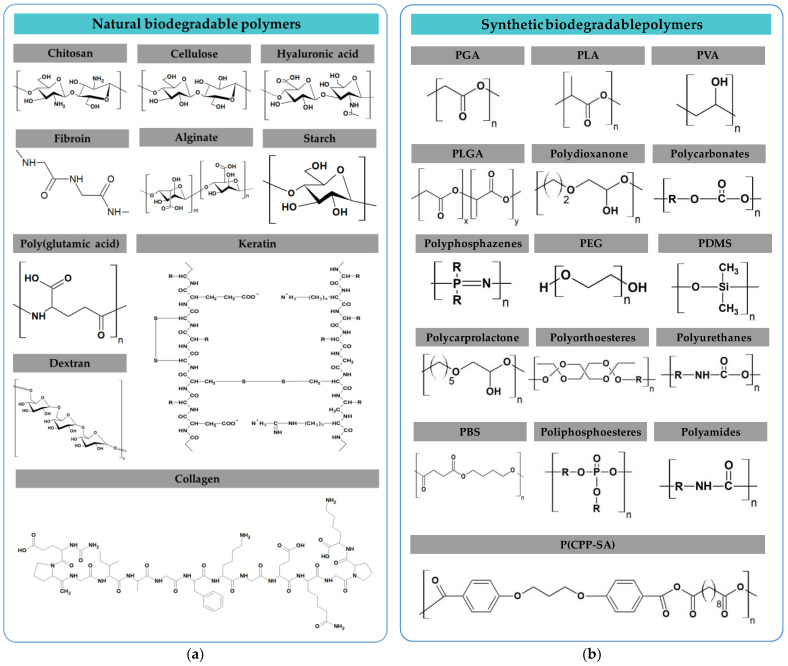
(**a**) Natural biodegradable polymers; (**b**) Synthetic biodegradable polymers.

Several types of biodegradable polymers can be found in nature, as shown in Figure 4a. These polymers exhibit dielectric properties. Due to their low dielectric loss and high voltage breakdown, natural polymers such as glucose, lactose, or adenine, among others, could be considered in the manufacture of biodegradable and biocompatible electronic devices. Likewise, biodegradable polymers could be synthesized, as shown in Figure 4b. As an advantage, these polymers’ physicochemical properties are more controlled, in comparison to natural ones, by controlling their synthesis conditions. Polymers such as polylactic acid (PLA), polyvinyl alcohol (PVA), poly(dimethyl siloxane) (PDMS), and polyurethane (PU) have been used in dielectric applications. The values of the dielectric constant of different biodegradable polymers are collected in Table 6.

Cellulose is a natural biopolymer found abundantly on Earth and is obtained from a vegetal source (annually, between 10^11^ and 10^12^ tons of nanocellulose are produced) [49]. The nanocelluloses are cellulose-based materials characterized by having nanoscale dimensions taken from plants, such as wood, coconut husk, sisal, algae, etc., or obtained from animals or bacteria [50,51]. They can be classified into three groups: (1) cellulose nanocrystals (CNCs), obtained chemically from plants or animals, (2) cellulose nanofibrils (CNFs), obtained mechanically, also from plants or animals, or (3) bacterial nanocellulose (BNCs), obtained from bacteria.

**Table 6:** Dielectric constants of potential biobased and/or biodegradable materials.

Material	Dielectric Constant	Frequency	Ref.
Cellulose	3.9–7.5	10^3^ Hz	[47]
Keratin	8	3 × 10^6^ Hz	[52]
Chitosan	5.5	10^3^ Hz	[53]
Starch	40–65	2.45 × 10^9^ Hz	[54]
Silk Fibroin	6.1	3 × 10^5^ Hz	[55]
Poly(glutamic acid)	130	10^3^ Hz	[56]
Hyaluronic acid	60	9 × 10^9^ Hz	[57]
Alginate	18.35	10^6^ Hz	[58]
Dextran	45–60	10^10^ Hz	[59]
Collagen	4.5	10^3^ Hz	[60]
PLA	2.9	10^3^ Hz	[61]
PVA	12	10^3^ Hz	[47]
Polycaprolactone	3	10^9^ Hz	[59]
PDMS	2.6	10^3^ Hz	[47]
PBS	17.5	-	[62]
Polyamides	3.5	10^3^ Hz	[63]
Polyethylene glycol (PEG)	11.6	10^3^ Hz	[64]
Polyurethanes	6–11	10^3^ Hz	[65]
Polycarbonates	3.0	10^3^ Hz	[47]

**Figure 5:**
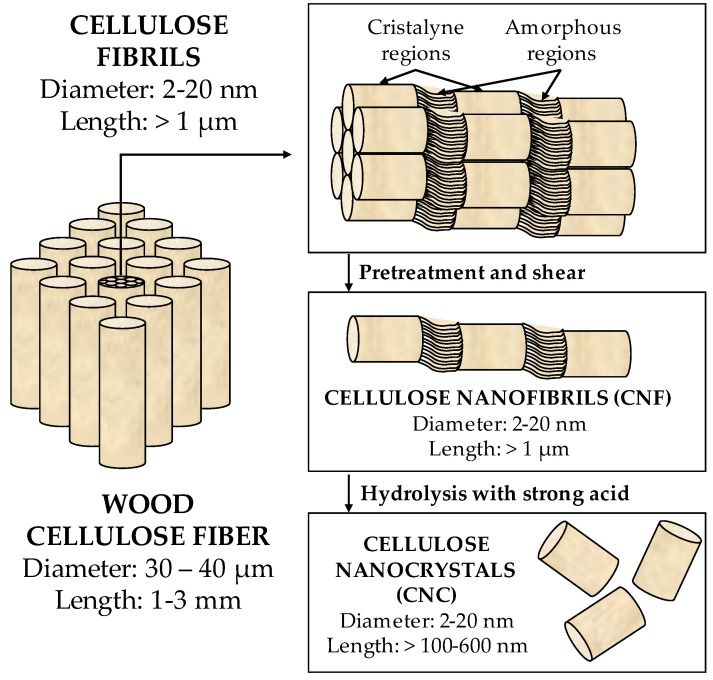
Manufacture of cellulose from wood cellulose fibers [51].

Figure 5 shows a scheme of the manufacturing of nanocellulose from wood cellulose fibers. CNFs present fiber morphology, with a wide range of lengths, between 0.3 µm and 10 µm, and diameters between 2 nm and 20 nm. CNCs have rod-shaped morphology, with diameters between 2 nm and 20 nm and lengths in the 100–500 nm range. The BNCs have the same structure as the CNCs and are produced by bacteria.

Cellulose is typically an insulating material whose dielectric properties are affected by its morphology. As an example, algae-containing nanocellulose, in comparison to wood-containing CNFs, has more crystallinity and less water sorption capability. Nevertheless, having more porosity, it has less electric resistance and more dielectric loss [66]. Thus, it is necessary to correctly select the cellulose type used in the formulation of a dielectric ink. Cellulose is resistant to hydrolysis due to its inter- and intra-molecular hydrogen bonds. For this reason, to ensure the biodegradability of cellulose, it is necessary to process it with microbes and fungal enzymes [67]. Williams et al. printed crystalline nanocellulose as a dielectric isolator [68].

Keratin protein is a protein that exhibits dielectric properties that can be used to produce green electronics. One source of keratin is chicken feathers, which are composed of 90% keratin. Singh et al. developed an aqueous keratin dispersion for dielectric coatings. King et al. developed various keratin aqueous dispersions, achieving dielectric constants up to 7.4 with frequencies of 10^6^ Hz [69].

Chitosan is a linear polysaccharide biopolymer, which also exhibits dielectric properties. Bonnard et al. developed biocomposites of chitosan and nitrile-modified cellulose nanocrystals, increasing its dielectric constant from 5.5 (pure chitosan) to 8.5 (composite with 50%wt.), measured at 10^3^ Hz [53,70].

Natural starches (such as tapioca, corn, wheat, or rice) also present dielectric properties. Ndife et al. prepared different starch dissolutions and measured their dielectric properties at 2.45 × 10^9^ Hz, obtaining a dielectric constant between 40 and 65 [54].

Allgén et al. studied the dielectric properties of sodium alginate in aqueous solution, obtaining dielectric constant values between 80 and 90, depending on the concentration of the sample [71].

Considering this, dispersions manufactured with the materials collected in Table 6 with dielectric properties are good candidates for developing sustainable inks for printed electronics.

#### 2.1.3. Piezoelectric Inks

Piezoelectricity is the property of certain materials to produce an electric current when suffering a deformation or to deform slightly when an electrical current is applied.

Piezoelectric materials could be classified into two distinct types: (1) materials of primordial piezoelectric nature (such as quartz) and (2) those known as ferroelectrics, which need a process of poling to present piezoelectric properties.

The most used piezoelectric materials are piezoelectric monocrystals, such as lithium niobate (LiNbO_3_) or lithium tantalate (LiTaO_3_), piezoelectric ceramics, such as barium titanate (BaTiO_3_) or lead titanate-zirconate (PZT), and piezoelectric composites, such as ceramic piezoelectric particles embedded in a polymer matrix.

Piezoelectric polymers have advantages over piezoelectric ceramics; they are weaker and can be cut into different shapes at low temperatures. Four classes of piezoelectric polymer materials are known: (1) polyvinylidene fluoride (PVDF) and its copolymers trifluoroethylene (TrFE) and tetrafluoroethylene (TFE); (2) distinct types of vinylidene cyanide copolymers (VDCN); (3) aromatic copolymers; and (4) aliphatic polyurea. These polymers are ferroelectric materials; it is necessary to apply a poling process to obtain their piezoelectric properties.

PVDF is the most well-known piezoelectric polymer and the one with the best possibilities due to its piezoelectric properties [72]. It is a semicrystalline polymer that exhibits distinct phases (α, β, γ y δ). At room temperature, α and β phases coexist; the β phase is responsible for the piezoelectric properties of the polymer [73]. The polymer needs a poling process to achieve piezoelectric properties. Ismail et al. demonstrated PVDF manufacturing from biobased and biodegradable carbonated solvents (ethylene carbonate (EC), propylene carbonate (PC), and butylene carbonate (BC)) [74]. However, the degradation of PVDF could produce harmful gases that damage the atmosphere and are harmful to human beings [67].

The quantity of piezoelectric inks available on the market is limited. While several companies sell conductive or dielectric inks, there are only a few companies that sell piezoelectric inks for printed electronics. These inks use P(VDF-TrFE) as a piezoelectric material. The Piezotech company’s inks are shown in Table 7 [75]. Nanopaint also produces an environmentally friendly piezoelectric ink, as shown in Table 7 [76]. The Magron company manufactures a piezoelectric ink, whose properties are shown in Table 7 [77].

Although wood’s piezoelectricity has been known for decades, the piezoelectric properties of CNFs and CNCs have only been studied during the last few years [78]. The piezoelectric responses measured in nanocellulose-based films are 2–8 pC/N.

Films based on plain chitosan exhibit piezoelectric properties with a sensibility of 4 pC/N. As with nanocellulose, it is necessary to apply a poling treatment to chitosan to develop its piezoelectric properties [70]. Chitosan is a biobased and biodegradable polymer and is becoming a good option for the development of piezoelectric inks.

Animal-based polymers are another material family showing piezoelectric properties. Two examples of these types of materials are silk, with a coefficient of up to 1 pC/N, and collagen, showing 2.64 pC/N. Both materials degrade enzymatically in physiologic conditions and in the presence of catalysts.

PVA is a water-soluble polymer known for its physicochemical properties, such as film formation, flexibility, and thermal stability [79]. There are studies showing a method for manufacturing high-quality crystalline thin layers of piezoelectric γ-glycine crystals between PVA layers. These thin films show a macroscopic piezoelectric response. The films are water-soluble, and, correctly packed, they could be used as part of an energy-harvesting biodegradable device [80]. PVA degrades under the presence and action of the phytopathogenic fungi *Fusarium lini*, producing carbon dioxide and water [81].

Poly (lactic acid) (PLA) is a biopolymer whose degradation occurs through hydrolysis in moist environments. This degradation could be accelerated with the addition of an enzyme such as bromelain or proteinase K. Using PLA, it is possible to manufacture films with piezoelectric behavior showing a sensibility between 3 and 5 pC/N. The measured sensibility, when flexed, is between 30 and 80 pC/N. This becomes a good option in applications requiring piezoelectric properties, especially when these properties are used in a flexion sensor [82].

Other biological materials with piezoelectric properties can be found in nature. As an example, 16 amino acids and their compounds hold piezoelectric properties at room temperature. Among them, the one with the most promising piezoelectric characteristics is γ-glycine, showing a coefficient of up to 10.4 pC/N. These responses could be compared with zinc oxide’s response, which could present a piezoelectric coefficient between 14.3 and 26.7 pC/N in specific conditions [67,83].

Another piezoelectric amino acid is DL-anime, whose coefficient is 10.34 pC/N, although it is decreased to 4 pC/N, on average, due to the random growth of the crystals [67,78]. Peptide also has piezoelectric properties that can be compared with perovskite [67]. These materials not only can be processed in a water solution, but they also decompose gradually, becoming basic molecules in water environments.

To obtain the piezoelectric properties of the mentioned materials, a poling process must be applied to them. This process consists, commonly, of the application of an electric field. This electric field rotates the dipoles present along the material in the direction of the applied electric field to obtain the piezoelectric properties [84]. Therefore, these types of materials are good candidates for developing piezoelectric sustainable inks, but it is necessary to process them correctly.

### 2.2. Polymeric Resin or Binder

**Figure 6:**
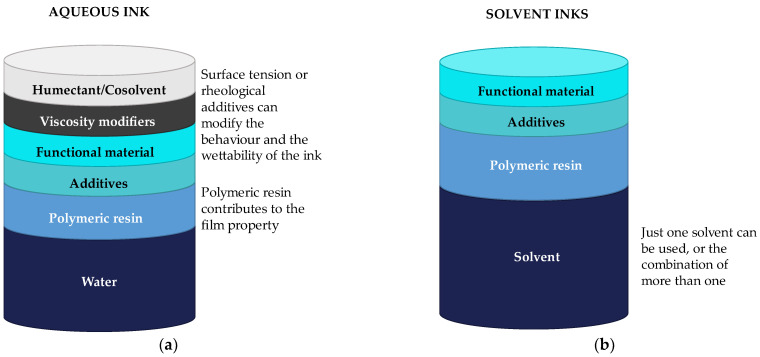
Scheme of the composition for (**a**) water-based inks and (**b**) solvent inks.

The binder is one of the essential parts of ink. Thus, it is necessary to select a biobased or biodegradable binder to formulate an ink. Depending on the selected dispersion media, the composition of the ink will vary. Figure 6 shows schematically the composition of water-based inks and solvent inks.

As presented above, nanocellulose is a biobased material. The biodegradability of functionalized nanocellulose has been demonstrated in certain conditions, and it is effective in various applications, including electronic applications [85]. It can be used both in the process of ink formulation for the synthesis of the nanoparticles (such as silver nanoparticles), acting as a template or capping agent, or as a stabilizer and binder. Brooke et al. developed a carbon nanocellulose-based ink, obtaining conductivities up to 4 × 10^2^ S/m [86]. Hoeng et al. manufactured a silver conductive ink in a CNCs suspension binder, obtaining conductivity when silver content is higher than 3% [87].

Other cellulose materials, such as cellulose acetate, are used as binders to fabricate carbon inks. Zappi et al. developed an ink with carbon particles obtained from lignin in a cellulose acetate binder [88]. This ink had an appropriate conductivity for manufacturing printed electrodes. Considering this, cellulose could be used to formulate biobased and biodegradable inks.

Dihydrolevoclucosenone (commercial name: Cyrene) is a biodegradable, non-mutagenic, and non-toxic solvent derived from biomass [89]. Pan et al. developed a Cyrene biobased ink with graphene particles, reaching conductivities of 7.13 × 10^4^ S/m [90]. The use of Cyrene as a binder avoids the use of other toxic solvents in the ink formulation because it is biobased and biodegradable.

Shellac is an organic resin secreted by a red insect living in Thailand [91]. It is a biopolymer from a natural and renewable source. Poulin et al. used shellac as a binder in the development of a screen printing conductive ink composed of carbon black particles, obtaining conductivities of 10^3^ S/m [2].

Water is an abundant, renewable, and biodegradable material on Earth, composing 70% of the planet. Hence, water-based inks are considered biodegradable whether ot not the rest of the materials fulfill the established rules. The patent US 2020/0339832 A1, published in 2020, describes the formulation of a conductive, sustainable ink with carbon particles extracted from hemp [92]. Rocha et al. developed a water-based ink with chitosan as the polymeric resin and graphite as the functional material [93]. Koga et al. prepared a carbon inkjet printer ink using CNTs and 2,2,6,6-tetramethylpiperidine-1-oxyl (TEMPO)-oxidized CNFs dispersed in water [94]. Martinez-Crespiera et al. formulated a water-based conductive ink with silver particles dispersed in nanocellulose [50].

Due to the capacity of PLA to degrade, it can be considered an appropriate binder for developing biodegradable ink for printed electronics. Different inks in PLA binders have been formulated [6]. For example, Atreya et al. developed an ink consisting of wolframium particles dispersed in PLA, obtaining a conductivity of up to 4.55 × 10^2^ S/m. The ink is water-resistant and biodegradable through hydrolysis or oxidation [6]. Najaf et al. manufactured printable conductive PLA-based inks using graphene in emulsion as the functional material. These inks presented a conductivity of 3.45 × 10^1^ S/m [5].

Polyethylene oxide (PEO) is a water-soluble polymer, used by Huang et al. to develop inks for electronic devices that solubilize when they are in contact with water [1]. The inks are composed of wolframium or zinc particles embedded in a PEO binder, and the conductivities measured were up to 4 × 10^4^ S/m.

Lee et al. elaborated on an ink using molybdenum particles as the functional material, dispersed in Poly butanedithiol 1,3,5-triallyl-1,3,5-triazine-2,4,6 (1H,3H,5H)-trione pentenoic anhydride. The obtained conductivities were near 1.4 × 10^3^ S/m for devices that degrade after 9 days in deionized water at 37 °C [95].

### 2.3. Solvent

The solvent is the part of the ink where the functional material is dispersed. As it is a fundamental part of the composition, it is important for it to be a biobased or biodegradable solvent (called eco-solvents), as well as compatible with the functional material and the selected binder.

Green solvents are environmentally friendly solvents, also called bio-solvents, derived from agricultural processes. As an example, ethyl lactate is a bio-solvent obtained from corn that is biodegradable and easily recycled. This solvent has been used in painting, substituting compounds such as toluene, acetone, or xylene, which are commonly used in inks. Furthermore, due to its solvency capability, it could be considered a good solvent for the development of sustainable inks [96].

Water can also be used as a solvent, obtaining a nanoparticle dispersion suitable for inkjet printing technology. For example, Mavuri et al. prepared a silver nanoparticle dispersion in water, propanol, and ethylene glycol used for inkjet printing [97]. Lin et al. dispersed CNTs in water, adding glycerol to achieve the viscosity needed [98]. Lee et al. sintered water-dispersed CNTs with methanol, which could be used for inkjet printing [99]. Other authors developed CNTs inkjet printing inks by dispersing the nanotubes in water [100,101,102].

Another bio-solvent could be natural polysaccharides obtained from bacteria, such as gellan or xantana, produced by the bacteria *Spingomomas eloda* and *Xanthomonas campestris,* respectively. Panhuis et al. developed water-based CNTs dispersed in these types of solvents [103].

**Figure 7:**
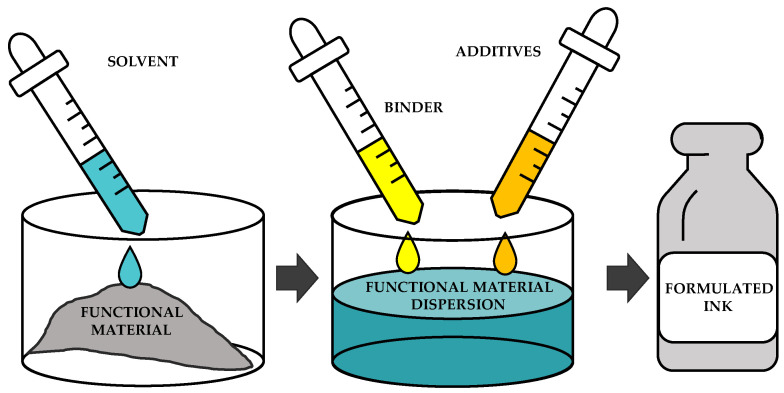
Scheme of the formulation of an ink.

To formulate the complete inks, it is necessary to select all the materials, ensuring compatibility between them as well as being able to keep the needed properties. As a general procedure to formulate inks, the functional material is dissolved in the solvent, and then the binder and the additives are added and mixed with an appropriate procedure. Figure 7 shows a scheme of this procedure.

All the biobased and/or biodegradable inks found in the literature, the materials used, their deposition method, and the reason for their sustainability are shown in Table 8.

## 3. Conclusions

**Figure 8:**
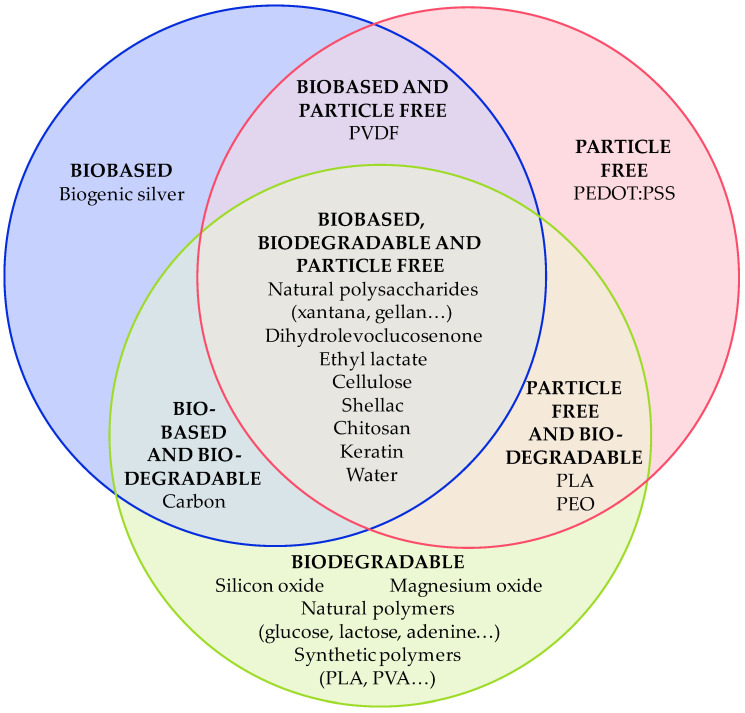
Classification of the materials used to develop sustainable inks, considering the proper material and quantity of each one to be considered biodegradable.

In this review, recent advances in the development of sustainable inks (conductive, dielectric, and piezoelectric) are presented. The sustainability of the inks can be achieved in three ways: (1) using biobased materials, (2) using biodegradable materials, or (3) avoiding the use of critical raw materials. The sustainability of the ink is secured by selecting the appropriate materials and considering the quantity of each material in the inks to be considered biodegradable. The classification of the different materials collected to develop inks for electronic applications is shown in Figure 8.

Electric conductive inks need a functional material that allows the electricity to pass through the printed lines. Three types of materials can be used: metallic materials, carbon-based materials, or polymeric materials.

Some metallic materials, such as silver or gold, despite presenting the highest conductivities (10^5^ S/cm), are considered critical raw materials and, therefore, are inappropriate for developing sustainable inks. However, there are studies that have developed a biobased silver, reducing the impact on the environment due to lower energy consumption in its synthesis and less toxic produced waste.

Carbon-based materials, such as graphite, graphene, carbon nanotubes, or carbon black, present conductivity (10^3^ S/cm) and can be used to develop sustainable inks. Carbon can be found in nature in multiple forms and is biobased and biodegradable. Taking all this into account, it is a good candidate for developing sustainable inks for printed electronics.

Other conductive materials that can be used are conductive polymers, such as PEDOT:PSS. Despite the fact that this material is neither biobased nor biodegradable, an ink with a low content of it could be considered sustainable and not harmful to the environment. Another strategy to develop a sustainable ink based on a conductive polymer is to disperse the PEDOT in a biopolymer, reaching conductivities of 10^1^ S/cm.

In printed electronics, dielectric inks are also needed to isolate parts of the circuitry or to protect devices from the natural environment, such as moisture. It is also necessary to develop sustainable inks that ensure these functionalities. Thus, a functional material with dielectric properties should be dispersed in a binder, such as cellulose or natural resins, or natural proteins, such as keratin. Different material dispersions have been reported and deposited on a substrate using different techniques, presenting suitable properties.

The last type of sustainable inks required are piezoelectric inks. There are biobased polymers (natural and synthetic) that present this property in particular conditions. Because of this, these materials could be dispersed in an appropriate binder. After the deposition, the materials must be exposed to a poling process to align all the dipoles of the material in the correct direction to obtain its piezoelectric properties. Currently, few piezoelectric inks have been found in the market or reported in the literature. By correctly selecting the materials and formulating an ink, it could be possible to develop a sustainable piezoelectric ink for printed electronics.

All these functional materials should be dispersed in a solvent and mixed with a binder. To be sustainable, these materials should also be biobased or biodegradable. Several types of binders and solvents have been found, such as water, PLA, cellulose dispersions, or resins coming from nature, such as shellac or xantana. The materials used must be compatible with the functional material selected, ensuring conductivity and good behavior to be printed using the method they are purposed to.

Different sustainable inks and dispersions have been found for screen printing, aerosol printing, and inkjet printing. The sustainability of these inks and/or dispersions depends on different parameters: the sustainability of the functional material, the sustainability of the binder, and the sustainability of the solvent.

To formulate the different sustainable inks, it is necessary to verify the compatibility between the different materials and to select them correctly. Moreover, it is necessary to define the deposition method and adjust the final properties of the formulated inks.

Currently, the development of biobased or biodegradable inks for printed electronics is a challenge. The inks must have good behavior and properties as well as competitive cost compared with commercial inks. Studies on developing this type of ink are being conducted. Another challenge that researchers may focus on is securing the recyclability of the ink and the global electronics systems in order to achieve sustainability objectives and green electronics.

## Figures and Tables

**Table 1:** 2023 European Union Critical Raw Materials.

2023 EU CRMs						
Antimony	Bismuth	Feldspar	Helium	Manganese	Phosphorus	Tantalum
Arsenic	Boron	Fluorspar	Heavy Rare Earth Elements (HREE)	Natural graphite	Platinum Group Metals (PGM)	Titanium metal
Bauxite	Cobalt	Gallium	Light Rare Earth Elements (LREE)	Nickel	Scandium	Tungsten
Baryte	Coking coal	Germanium	Lithium	Niobium	Silicon metal	Vanadium
Beryllium	Copper	Hafnium	Magnesium	Phosphate rock	Strontium	

**Table 2:** Ink composition.

Material	Screen Printing	Inkjet Printing	Refs.
Functional material	5–70%	10–30%	[13,14]
Polymeric resin/Binder	20–50%	5–30%	[13,14]
Solvent	15–65%	60–90%	[13,14]
Additives	<5–10%	<5–10%	[13,14]

**Table 3:** Conductivity of some functional materials used in conductive inks.

Material	Conductivity	Ref.
Silver	6.8 × 10^7^ S/m	[20]
Copper	5.98 × 10^7^ S/m	[20]
Gold	4.3 × 10^7^ S/m	[20]
Aluminium	3.8 × 10^7^ S/m	[20]
Magnesium	2.2 × 10^7^ S/m	[21]
Wolframium	1.8 × 10^7^ S/m	[22]
Zinc	1.7 × 10^7^ S/m	[21]
Nickel	1.5 × 10^7^ S/m	[20]
Iron	1.04 × 10^7^ S/m	[21]
Platinum	9.5 × 10^6^ S/m	[21]
Palladium	9.4 × 10^6^ S/m	[21]
Tin	8.7 × 10^6^ S/m	[21]
Carbon	10^2^–10^6^ S/m	[23]
PEDOT: PSS	2 × 10^−1^–2.1 × 10^5^ S/m	[23]

**Table 4:** PEDOT:Biopolymer dispersions and achieved conductivities.

PEDOT:Biopolymer	Conductivity (S/m)	Ref.
PEDOT:dextran sulphate	7 × 10^2^	[42]
PEDOT:DNA	10^2^	[42]
PEDOT:heparin	0.1–5	[42]
PEDOT:chondroitin Sulphate	0.2–7.5	[42]
PEDOT:hyaluronic acid	0.3–7.1	[42]
PEDOT:sulphated cellulose	57.6	[42]
PEDOT:pectin	<1	[42]
PEDOT:guar gum	2.8–12.9	[42]

**Table 5:** Dielectric constants of materials used in electronics.

Material	Dielectric Constant	Frequency	Ref.
Paper	2–4	10^6^ Hz	[43]
Mica	3–6	10^3^ Hz	[44]
Teflon	2	10^3^ Hz	[45]
Rubber	6.7	-	[46]
Polymers (general)	~2	10^3^ Hz	[43]
High-Density Polyethylene (HDPE)	2.3	10^3^ Hz	[43]
Low-Density Polyethylene (LDPE)	2.3	10^3^ Hz	[43]
Polypropylene (PP)	2.3	10^6^ Hz	[43]
Mylar	3.25	10^3^ Hz	[43]
Kapton	3.9	10^3^ Hz	[43]
Polyvinyl chloride (PVC)	3.4	10^3^ Hz	[47]
Glass (Pyrex)	5	-	[46]
Porcelain	6–8	-	[46]

**Table 7:** Piezoelectric commercial inks.

Company	Name	Technology	Material
Piezotech^®^—Arkema-CRRA (Pierre-Bénite, France) 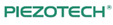	Ink L	Spin-coating Slot-die	Piezotech^®^ FC20 Piezotech^®^ RT-TS
	Ink H	Spin-coating Solvent-casting	Piezotech^®^ FC20 Piezotech^®^ RT-TS
	Ink P	Screen printing	Piezotech^®^ FC20
			Piezotech^®^ RT-TS
Nanopaint (Braga, Portugal) 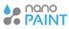	PEInk01NP	Screen printing	PVDF-TrFe
Magron (Ansan-si, Republic of Korea) 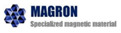		Screen printing Doctor blade Stencil Spray	PVDF-TrFe

**Table 8:** Materials and conductivity of biodegradable inks for electronics.

Binder	Active Element	Solvent	Other Materials			Tech.	Sustainability Reason	Ref.
PEO (Polyox N-80)	Zinc// Wolframium	Methanol	-			Screen printing	Biodegradable binder	[1]
Poly butanedithiol triallyl isocyanurate	Molybdenum (1–5 µm)	PBTPA	2,2-dimethoxy-2- phenylacetophenone			Screen printing	Biodegradable binder	[95]
Cyrene	Graphene	N-methyl-2- pyrrolidinone (NMP, >99%)	CAB (butyryl content 35–39%)			Screen printing	Biodegradable binder and graphene as functional material	[90]
PLA	Wolframium	Tetrahydrofuran (THF)	-			Screen printing	Biodegradable binder	[86]
PLA	Graphene nanoplates (GnPs)	Anisole (C_7_H_8_O)	Sodium carboxymethyl cellulose (SCC)			Screen printing	Biodegradable binder and graphene as functional material	[5]
			Polyoxyethylenesorbitan Tristearate (Tween^®^ 65)					
			Milli-Q ultrapure water					
Shellac	Graphite flakes	Ethanol/ Pentanol	Carbon black			Screen printing	Biobased and biodegradable binder and graphite as functional material	[5]
			PEG					
Nanocellulose	Carbon black	Propylene glycol	Glycerol			Screen printing	Biobased binder and carbon black as functional material	[86]
			2-HEC					
Cellulose acetate	Carbon from lignin	Cyclohexane				Screen printing	Biobased carbon and biodegradable binder	[88]
Chitosan	Graphite	Water based: Acetic acid aqueous solution	Glycerol			Screen printing	Graphite as functional material and water-based	[93]
Gellan gum// Xantana gum	CNTs	Water-based	-			Inkjet printing	Carbon as functional material, biobased and biodegradable solvent and water-based	[103]
Aqueous dispersion TEMPO -oxidized CNFs	CNTs	Water	-			Inkjet printing	Carbon as functional material, water-based and nanocellulose as binder	[94]
CNCs suspension	Silver	CNCs suspension	Hexadecylpyridinium chloride solution			Inkjet printing	CNCs as the binder	[87]
			AgNO_3_					
			NaBH_4_					
Aqueous solution of TEMPO	Silver	Water-based: Water/ Isopropyl alcohol	CNCs	NaBr	Ethanol	Screen printing	Water as solvent and cellulose as additive	[50]
			NaOCl	NaOH	AgNO_3_			
			NaBH_4_ or Hydrazine					
			Ethylene glycol					
			Dispersing agent (Disperbyk 2012 1–5 wt.%)					
			Hydroxypropyl methylcellulose					
			Rheological additive (Reobyk 7420)					
			HCl					
-	PEDOT: dextran sulfate	Water-based	-			Inkjet printing	Biopolymer to increase PEDOT conductivity and water-based	[42]
-	PEDOT: DNA	Water-based	-			Inkjet printing	Biopolymer to increase PEDOT conductivity and water-based	[42]
-	PEDOT: heparin	Water-based	-			Inkjet printing	Biopolymer to increase PEDOT conductivity and water-based	[42]
-	PEDOT: chondroitin sulfate	Water-based	-			Inkjet printing	Biopolymer to increase PEDOT conductivity and water-based	[42]
-	PEDOT: hyaluronic acid	Water-based	-			Inkjet printing	Biopolymer to increase PEDOT conductivity and water-based	[42]
-	PEDOT: sulfated cellulose	Water-based	-			Inkjet printing	Biopolymer to increase PEDOT conductivity and water-based	[42]
-	PEDOT: pectin	Water-based	-			Inkjet printing	Biopolymer to increase PEDOT conductivity and water-based	[42]
-	PEDOT: guar gum	Water-based	-			Inkjet printing	Biopolymer to increase PEDOT conductivity and water-based	[42]
-	Sodium Alginate	Water-based	-			Screen printing	Alginate as functional material and water-based	[71]
-	CNCs	Water-based	-			Aerosol printing	CNCs as active material and water-based	[68]

## Data Availability

No new data were created or analyzed in this study. Data sharing is not applicable to this article.
